# Personalised Medicine: The Odyssey from Hope to Practice

**DOI:** 10.3390/jpm8040031

**Published:** 2018-09-21

**Authors:** Sophie Visvikis-Siest, Vesna Gorenjak, Maria G. Stathopoulou

**Affiliations:** 1 Inserm, IGE-PCV, Université de Lorraine, 54000 Nancy, France; gorenjak.vesna@gmail.com (V.G.); maria.stathopoulou@inserm.fr (M.G.S.); 2 Department of Internal Medicine and Geriatrics, CHU Technopôle Nancy-Brabois, Rue du Morvan, F-54511 Vandoeuvre-lès-Nancy, France

**Keywords:** personalised medicine, pharmacogenomics, ethics, omics, Odyssey

## Abstract

In this endeavour, inspired by the Odyssey, we aim to embark with the reader on a journey on a ship from Troy to Ithaca, coursing through the history of the momentous events and achievements that paved the way for personalised medicine. We will set sail amidst important genetic discoveries, beginning with the discovery of the first human genome, and voyage through the projects that contributed to the progress of pharmacogenomic studies. Concurrently, we will propose methods to overcome the obstacles that are slowing the potential full implementation of accumulated knowledge into everyday practice. This journey aims to reflect on the frontiers of current genetic knowledge and the practical use of this knowledge in preventive, diagnostic and pharmacogenomic approaches to directly impact the socio-economic aspects of public health.

## 1. The Journey from DNA Structure to Personalised Medicine

“*Tell me, O Muse, of that ingenious hero who travelled far and wide after he had sacked*

*the famous town of Troy. Many cities did he visit, and many were the nations with*

*whose manners and customs he was acquainted…*” [1]

Decades ago, the study of genetics was similar in ardour and endurance to the conquering of the famous city of Troy, described in Homer’s epic poem, “The Iliad”. Every story has its heroes, and at the end of the 1950s, the heroes in genetics were James D. Watson and Francis Crick [2], the most well-known scientists related to the discovery of DNA structure and function. “*The horse has entered the fortress and the battle began*.” [1].

Since then, important findings followed one another: the DNA code was determined [3], and transcription [4], translation [5] and replication [6] were described. Laboratory techniques required for such research rapidly developed and computer science became more accessible. When Frederick Sanger presented a new method for DNA sequencing [7], for which he was awarded the Nobel prize in chemistry in 1980 [8], understanding the genetic code became even easier and, thus, expectations rose. The next goal for scientists was genotyping the entire human genome.

The ambitious Human Genome Project (HGP) was launched in 1990 under the direction of the U.S. National Center for Human Genome Research, which is currently known as the National Human Genome Research Institute (NHGRI) [9]. The sequencing of three billion base pairs was performed in collaboration with 20 groups from the USA, the UK, Japan, France, Germany and China and was completed 13 years later, in April 2003 [10]. The HGP provided vast and comprehensive catalogues of genomic information on biological structure and function and allowed the investigation of basic genome physiology. Most importantly, it was a landmark in medical research.

Constituting a new focus of interest for scientists were the millions of single nucleotide polymorphisms (SNPs), which represent differences in single DNA building blocks [11]. Indeed, millions of small variations in the human genome lead to different disease and treatment susceptibilities. However, relating SNPs to a disease demands extensive methodologies that require high technological standards.

Despite the difficulty of categorising the complex web of environmental factors that also contribute to inter-individual variability, improvements in genomics research permitted the characterisation of genetic variants that account for this variability to a large extent. Due to the early initiation of the HGP, progress in genotyping and sequencing techniques was rapid. The facilitation of gene identification made exploring the genetic background of diseases a new common practice and provided a flood of new information that led to tremendous progress in understanding the pathophysiological processes of diseases [12], thereby ushering in personalised medicine.

The initial intent of personalised medicine was to collect “-omics” data to provide new information about the mechanisms underlying disease and to thus identify new strategies for prediction, prevention and treatment to ensure personalised and participatory healthcare [13]. Although personalised medicine first appeared as a scientific term in published studies in 1991 [14], the concept originated long ago in the history of medicine, when scientists realised that certain treatments did not apply to all patients and might be harmful in some cases. One of the best examples is blood transfusions. The ABO blood group system was discovered in 1900 [15] and such knowledge helped prevent complications during transfusion through a simple blood type test that categorised each individual into different groups based on the ABO genotypes. This simple illustration demonstrates well the mission of personalised medicine today, which is to classify patients into different subgroups based on their genetic predispositions to allow for early disease risk prediction, precise diagnosis, accurate prognosis and the use of the most efficient and least harmful treatments for each individual.

Because of the very broad and general meaning of the term “personalised” medicine, some scientists and clinicians refer to it as “precision medicine” to emphasise the primary aspect of the field, which concerns targeted treatments constructed on the basis of the genetic, phenotypic and psychosocial characteristics of an individual patient, through which such an individual differs from other patients with the same condition [16]. We prefer the term “personalised” medicine because of the broadness of the expression, which refers to all important domains of the field.

**Figure 1:**
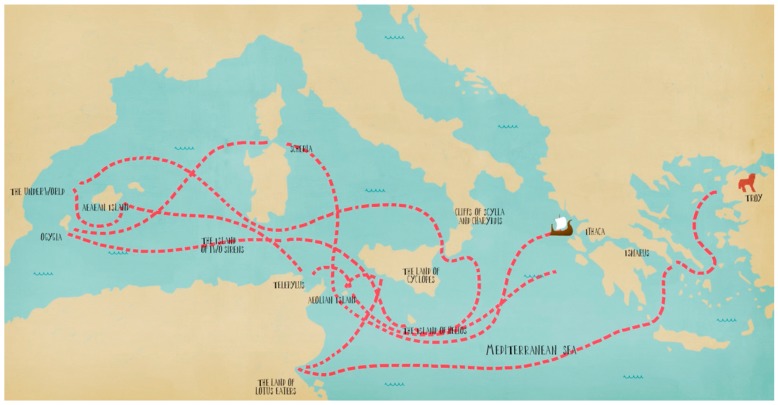
The journey of Odysseus. 0: Troy; 1: Ismarus; 2: The land of the Lotus-eaters; 3: The land of the Cyclopes; 4: The Aeolian island; 5: Telepylus, the city of the Laestrygonias; 6: Aeagean island (Circe’s island); 7: The Underworld (The house of Hades); 8: The island of the two Sirens; 9: Cliffs of Scylla and Charybdis; 10: The island of Helios; 11: Ogygia (Calypso’s island); 12: Scheria (island of the Phaeaians); 13: Ithaca.

This article explores the research journey towards personalised medicine by connecting it to the adventurous trip of Odysseus, from Troy to Ithaca (Figure 1). His journey might have seemed sorrowful at the first glance, but above all it was rewarding and meaningful. Hence, it is in personalised medicine, where while fighting numerous obstacles and deceptions we finally end up realising remarkable discoveries and projects, which will greatly contribute to the next generation of health systems.

## 2. First Stop: Ismarus, the City of the Cicons and the Difficulties of Genomic Research

“*When I had set sail thence the wind took me first to Ismarus, which is the city of the*

*Cicons. There I sacked the town and put the people to the sword. We took their wives*

*and also much booty, which we divided equitably amongst us, so that none might have reason to complain*.” [1]

The journey of personalised medicine began with the launching of the HGP, which commenced at a time when the general public was beginning to become familiar with intriguing genetic discoveries, as governments invested in “prospective studies” within this new emerging field. Much optimism and excitement accompanied the beginning of the “-omics” era, and many scientists were eager to participate, just like Odysseus and his soldiers after the Trojan war were excited to embark on the journey that would take them home after a long and exhausting battle.

The initial idea of personalised medicine was extremely ambitious. However, progress was not as fast as expected. Most common chronic diseases appeared to be regulated by a complex mixture of genetic factors and an infinite number of environmental factors, which were more difficult to elucidate than initially expected. Expensive “-omics” studies showed some success, though the clinical applications were few. Hence, criticism began to ensue regarding genomic research [17]. Questions arose such as whether the associated thought processes were all in error from the beginning, whether the money spent on research and development might never pay off and whether greed for success had derailed some researchers from their goals.

*After the great victory in Troy, Odysseus, the great hero of the war, stopped on his way home on the island of Ismarus and, drunk of success, made a fatal mistake of letting his men destroy a town. Thus, the angry gods called the winds that misled his ships and took him on a long and dangerous journey that would detain him from reaching his own goal, Ithaca*.

## 3. Second Stop: The Lotus-Eaters and the International HapMap Project

“*I was driven thence by foul winds for a space of nine days upon the sea, but on the*

*tenth day we reached the land of the lotus-eaters, who live on a food that comes from a kind of flower*.”

“*… so delicious that*

*those who ate of it left off caring about home, and did not even want to go back and say what had happened to them, but were for staying and munching lotus with the*

*lotus-eaters without thinking further of their return …*” [1]

The International HapMap Project represented the first substantial achievement in the determination of common genetic patterns of DNA sequence variation, which allowed for the identification of the sequence variants that affect common diseases and their frequencies and correlations. The project began in October 2002 as an international collaboration and was a natural extension of the HGP [18]. Whereas the HGP focused on the genome, which is invariant across individuals, the project’s haplotype mapping (HapMap) focused on DNA sequence differences among individuals.

Common genetic patterns of DNA sequence variation, called haplotypes, are specific combinations of alleles on a chromosome. This combination is predictable because individuals who carry a specific SNP allele at one site often carry specific alleles at other nearby variant sites. This observation indicates that neighbouring SNPs on the same chromosome are in so-called linkage disequilibrium (LD) [19].

The HapMap of the human genome mapped and correlated SNPs based on their LD, which thus provided the information needed to guide genetic studies on clinical phenotypes. Phase I of the project, completed in 2005, provided publicly available data of the human genome sequence, databases of common SNPs, insights into human LD, web-based tools for storing and sharing data and frameworks to accelerate the identification of the genetic factors that influence medical traits. More than 1.1 million SNPs of 270 individuals across different global populations were genotyped during the project, which had a central role in developing the methods for designing and analysing genome-wide association studies (GWAS). The complete characterisation of SNP variation and LD among common variants in the sampled populations led to an excellent understanding of genetic variation in the human population and allowed for the determination of the haplotypes that might be involved in specific diseases [19,20,21,22].

Phase II of the project characterised an additional 2.1 million SNPs in the same individuals, which thus achieved the goal of a SNP density of approximately one SNP per kilobase. Phases I and II of the International HapMap Project genotyped approximately 25–35% of the total 9–10 million common SNPs in the assembled human genome, with a minor allele frequency (MAF) of >0.05 [21]. The project was completed with the release of the Phase III dataset in spring 2009, for which 1.6 million SNPs were genotyped in 1184 reference individuals to generate an integrated dataset of common and rare alleles [23].

Combined with the HGP and the SNP consortium, the International HapMap Project contributed to the identification of approximately 10 million common DNA variants and the understanding of their LD patterns, which enabled GWAS that identified thousands of newly discovered genes related to diseases [23]. The International HapMap Project was the first considerable achievement after the HGP. It greatly contributed to the development of personalised medicine studies and provided tools that served scientists for a decade.

**Figure 2:**
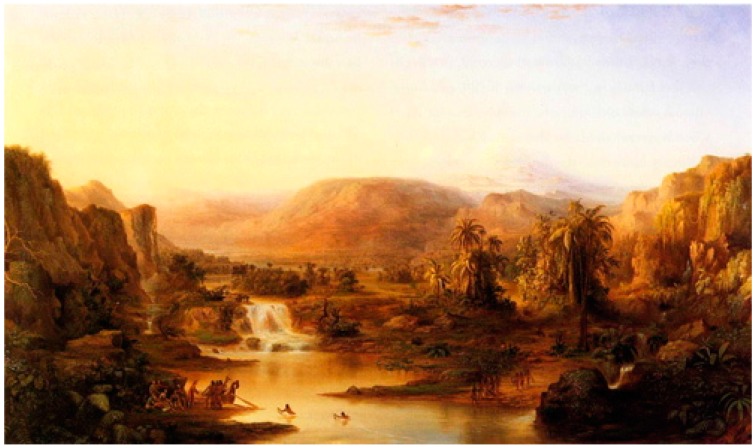
*Land of the Lotus Eaters*: Robert Duncanson, 1863.

*The achievements or feelings that we once found extraordinary and unforgettable sink into oblivion when our attention is drawn to new attractive things. In 2016, the National Centre for Biotechnology Information (NCBI) retired the HapMap dataset and website, which were overshadowed by new monumental research, including the 1000 Genome Project [24]. A similar shift occurred with some of Odysseus’s soldiers after the second stop on the island of the Lotus-eaters. Seduced by the sweet elixir of the lotus flower, they forgot the victory they achieved in Troy and their craving to see the homeland. Their only interest became the lotus flower, so they decided to stay on the island and never return to Ithaca. Thus, Odysseus chose to depart with the rest of his crew to continue towards new victories and left them behind on the island of the Lotus-eaters (Figure 2)*.

## 4. Third Stop: The Land of Cyclopes and Pharmacogenomic Studies

“*We sailed hence, always in much distress, till we came to the land of the lawless and*

*inhuman Cyclopes. Now the Cyclopes neither plant nor plough, but trust in providence,*

*and live on such wheat, barley, and grapes as grow wild without any kind of tillage, and their wild grapes yield them wine as the sun and the rain may grow them*.”

“*When the child of morning, rosy-fingered Dawn appeared, we admired the island and*

*wandered all over it …*” [1]

Traditional medicine, along with the pharmaceutical industry, has been successfully saving lives for decades. However, with the sometimes surprising number of possible side effects listed on medication packages, we are left hoping to not be one of the “unlucky users”. Individuals have different ways of responding to an administered drug, which is conditioned by their genetic predisposition. Indeed, small inherited variations in a single gene may affect a patient’s susceptibility to specific drugs. These considerations were not accounted for during traditional drug design. Hence, currently, some of the highest-grossing drugs are effective for as few as 1 of 25 to 1 of 4 patients [25]. The latest meta-analysis on the prevalence of medication-related adverse events among inpatients in the Western world estimated that adverse drug events affect 19% of patients during hospitalisation [26]. Furthermore, more than 4% of urgent hospitalisations are estimated to be caused by adverse drug events, with age and polypharmacy among the primary risk factors [27], which signify a large burden for patients and health systems.

Pharmacogenomics studies represent the basis for the clinical application of personalised medicine. The study of the impact of genetic variations on drug development is leading to the development of therapies that might ensure the best response and highest safety for a patient, whilst preventing or minimising the risks of side effects. The goal of pharmacogenomic research is to provide effective therapies for smaller subgroups of patients who are stratified based on their genetic profile, despite having similar disease phenotypes. Moreover, these studies aim to prevent adverse effects using preliminary genetic tests to determine whether patients might benefit from the drug and will allow dose adaptation for each patient based on their metabolic system characteristics. Finally, they will lead to the development of specific drugs for patients who are unresponsive to available treatments [28,29].

Pharmacogenetics is a subgroup of the pharmacogenomics field that focuses on examining genetic variations in drug metabolism [30]. Although bearing enormous potential, the translation from discoveries to clinical applications remains slower than desired [31]. In contrast, its successful applications increase hope and optimism and drive change for the future of practice in all aspects of personalised medicine.

Firstly, the case of abacavir demonstrated that through genetic examination, the response to a specific drug therapy might be predicted [32]. A severe adverse effect (abacavir hypersensitivity syndrome) manifested in 4–8% of patients treated with this anti-human immunodeficiency virus (HIV) drug. This reaction was later related to the major histocompatibility complex (MHC) class I allele HLA-B*5701, with a negative predictive value of 100%. Currently, the HIV treatment guidelines strongly recommend HLA-B*5701 testing prior to using abacavir [33,34]. Another example of drug effectiveness prediction using a personalised approach is gefitinib, a cancer drug prescribed for metastatic non-small-cell lung cancer, approved by the U.S. Food and Drug Administration (FDA) in 2003. Treatment with this therapy was only successful in 40% of patients. A year later, research groups demonstrated that these 40% of patients had mutated *EGFR* genes. This discovery allowed for the development of personalised lung cancer treatments using a new generation of targeted drugs [35,36,37].

Secondly, the testing of leukaemia patients for the thiopurine *S*-methyltransferase (*TPMT*) gene is a practical use of genetic testing to determine the ideal drug dose. *TPMT* is a gene responsible for producing an enzyme that metabolises thiopurines. A decreased ability to metabolise the drug was observed in 10% of the patients, due to a genetic change that reduces the number of high-activity copies. For these patients, a reduced thiopurine dose prevents undesirable effects and allows for successful therapy [38,39]. In fact, several known genes in addition to *TPMT* exist that are responsible for the variance in drug metabolism, specifically the cytochrome P450 gene family (*CYP*), vitamin K epoxide reductase complex subunit 1 (*VKORC1*), dihydropyrimidine dehydrogenase (*DPYD*) and uridine diphosphate glucuronosyltransferase (*UGT*) [40,41]. Whilst pharmacogenetic tests for these genes are indispensable for the correct pharmacokinetic calculations and drug dosages for patients, they are not fully implemented in daily clinical practice. Another example of drug dose adaptation aided by pharmacogenomics is the case of warfarin, a vitamin K antagonist used as an anticoagulant [42,43]. The FDA recommended genetic testing for *VKORC1* and *CYP2C9* polymorphisms, which account for 25–35% of the variance in warfarin dosing [44]. This testing is challenging because it is costly, inadequately available and time consuming [43]. However, additional studies are needed to incorporate genetic testing strategies into drug dose regulation in the future.

The first FDA-approved pharmacogenetic test was released onto the market in 2005. The AmpliChip™ CYP450 Test (Roche Molecular Systems, Inc.) is indicated to assess the metabolism rate of drugs metabolised by the CYP 2D6 and 2C19 isoenzyme, based on the genotyping of 27 alleles in *CYP2D6* and 3 alleles in *CYP2C19* genes associated with different metabolic phenotypes [45]. Currently, many more genetic tests are available to optimise drug therapy. Guidelines and tables of the available tests are presented on the Clinical Pharmacogenetics Implementation Consortium website [46].

**Figure 3:**
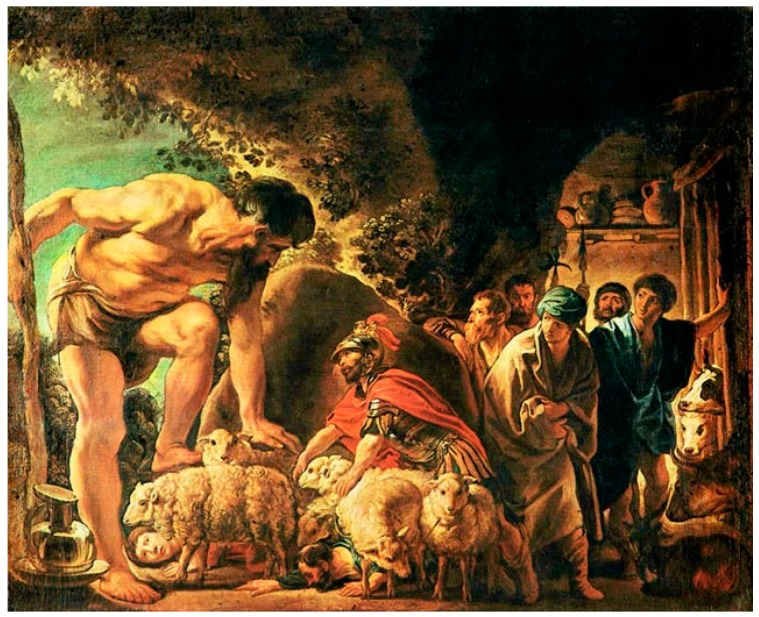
Odysseus in the Cave of Polyphemus: Jacob Jordaens, 1635.

*We are still in the land of the Cyclopes, where many events have transpired since Odysseus left his ship and went exploring the unknown. Trapped in a cave with Polyphemus, son of Poseidon, who ate some of his men, Odysseus was like the researchers, trying to use all their creativity to escape the undesirable events. He blinded the eye of the giant Cyclope, hid himself and the rest of the men under the sheep (Figure 3), and thus provided himself a safe escape from the island. His group of soldiers survived, because blind Cyclope could not recognise them, as he would, if they would not be attached to the animals. And that is the simple principle of the pharmacogenomics treatment*.

## 5. Fourth Stop: Aeolian Island and Genetic Association Studies

“*It is an island that floats (as it were) upon the sea, iron bound*

*with a wall that girds it. Now, Aeolus has six daughters and six lusty sons, so he made*

*the sons marry the daughters, and they all live with their dear father and mother,*

*feasting and enjoying every conceivable kind of luxury*.”

“*He [Aeolus] put the sack in the ship and bound the mouth so*

*tightly with a silver thread that not even a breath of a side-wind could blow from any*

*quarter. The West wind, which was fair for us, did he alone let blow as it chose; but it all came to nothing, for we were lost through our own folly*.” [1]

Genetic association studies are used to test the association between a specific disease (specific phenotype) and genetic variations to identify candidate genes or regions within the genome that might contribute to that disease [47]. The most widely tested genetic variations are SNPs, which are either assessed in candidate gene research [48] or through the more recently popularised GWAS [49]. Whilst candidate gene research confirms or refutes the correlation between a priori selected specific genetic variants and a disease, GWAS provides tests without a hypothesis for the specific regions, genes, or variants, albeit with a pre-selected design for genotyping platforms and analytical methodologies [50]. GWAS enabled the efficient and comprehensive assay of genetic variants that are common in a population and the identification of those that appear more commonly in patients with a given condition [51].

Although candidate gene studies brought remarkable success to the identification of high relative risk genes, they have not been as successful in the identification of genes involved in the complex forms of diseases [48]. Thus, the first GWAS, published in 2005 [52], generated enthusiasm among researchers.

**Figure 4:**
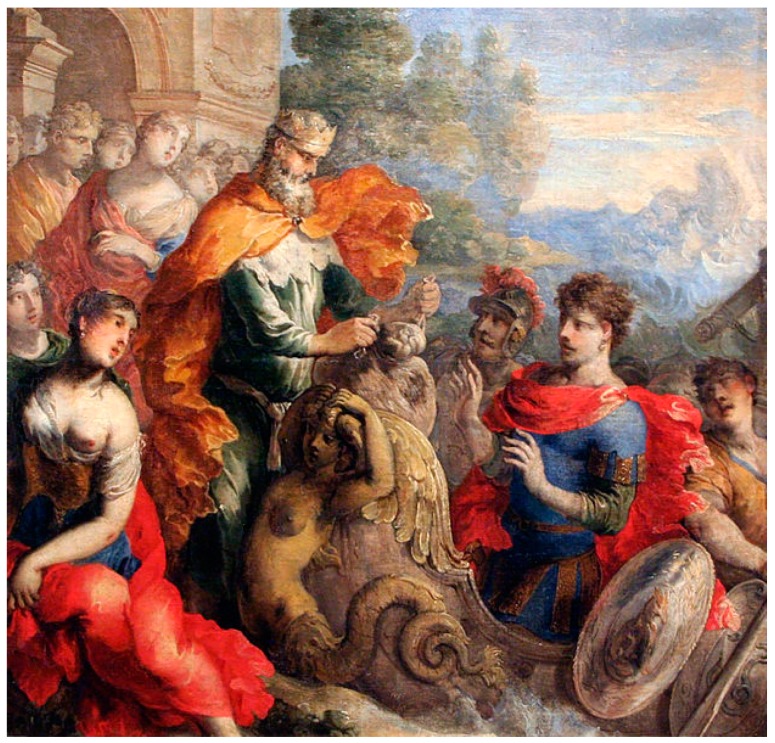
Aeolus Giving the Winds to Odysseus: Isaac Moillon, unknown date. Receiving a remarkable gift is of little value if it is not used appropriately.

*Before leaving the Aeolian island and sailing towards his homeland, Odysseus received a special gift from the god of winds (Figure 4)—a tightly bounded sack. None of his soldiers was aware of its contents, and they became curious, believing that Odysseus was hiding treasures from them*.

“*Nine days and nine nights did we sail, and on the tenth day, our native land showed on the horizon*.” [1]

*Soon before reaching Ithaca, they opened the sack. The winds that were trapped inside started to blow again and took their ship back to the Aeolian island*.

If GWAS at the beginning seemed like a gift, which will give the solutions to all problems, the high expectations were soon extinguished. These studies demanded a large number of samples for adequate statistical power [53]. Indeed, studies with hundreds of thousands of participants might only explain a small proportion of overall heritability. For example, significantly associated common variants in type 2 diabetes appear to merely explain approximately 6% of the increased risk of disease among relatives [54]. The number of genes related to complex diseases each accounted for only up to 10–30% of the genetic component of any given trait, thus indicating a new challenge, the so-called “hidden heritability” [55].

Nevertheless, GWAS connected thousands of novel genetic variants to many complex diseases, including diseases with no previously known genetic linkage. Their limitations, such as statistical strength and precision, were overcome using the statistical tool of meta-analysis, which combines the results of different genetic association studies to explore different sources of heterogeneity and to identify subgroups associated with the factor of interest [56]. Furthermore, the latest GWAS studies that are performed in very large populations, the improvements of computational tools, and the development of large datasets such as the UK Biobank provide new insights for the utility of GWAS studies. Recently, polygenic scores derived from GWAS studies have been shown to be as useful as monogenic mutations for the prediction of chronic diseases, thus opening a new direction for the application of GWAS results in clinical practice [57]. The next important step is to match these results to a larger biological construct to unravel the most complicated pathways related to a disease and to thereby contribute knowledge that might be translated into clinical and diagnostic tools [55].

*Similarly, Odysseus and his crew had to take the next step and find their own way further on after the sorrowful event, for Aeolus, the god of winds, wished no more to help them*.

## 6. Fifth Stop: The Laestrygones and Prediction Tools of Personalised Medicine

“*Six days, night and day did we toil, and*

*on the seventh day, we reached the rocky stronghold of Lamus—Telepylus, the city of*

*the Laestrygonians, where the shepherd who is driving his sheep and goats [to be*

*milked] salutes he who is driving out his flock [to feed] and answers the*

*salute. In that country, a man who could do without sleep might earn double wages, one*

*as a herdsman of cattle, and another as a shepherd, for they work much the same by*

*night as they do by day*.” [1]

Another important aspect of personalised medicine, in addition to predicting the response to a treatment or adapting the drug dosage (discussed in the third stop of the journey), is predicting the likelihood of developing a specific disease. This ability may be indispensable in specific families with disease antecedents, wherein rapid discovery by genetic testing might prevent the onset of the disease. The famous case of actress Angelina Jolie raised awareness of these methods among the general population, when she announced she was undergoing a risk-reducing mastectomy after testing positive for the *BRCA1* gene mutation [58], a major risk factor for breast cancer. Six months after the announcement, the demand for genetic tests increased by more than 100% [59].

Currently, more than 2000 genetic tests are available for several different indications [60] to:

- identify genetic diseases in unborn babies
- determining whether people carry a disease gene and might pass it on to their children
- screen embryos for disease
- test for genetic diseases in adults before they are symptomatic
- diagnose a person who has disease symptoms
- determine the type or dosage of a medication that is optimal for a specific individual

The Genetic Testing Registry (GTR^®^), part of the National Center for Biotechnology Information (NCBI) (available at: https://www.ncbi.nlm.nih.gov/gtr/), provides detailed information on all available genetic tests for a condition or drug response.

Genetic testing is voluntary. A positive result may guide a person towards available prevention, monitoring, and treatment options. In some cases, although no solution might exist for the given condition, genetic tests may aid decision-making concerning family planning [60]. Science is diligently attempting to improve the potential for gene therapies that might prevent the diseases caused by gene mutations. In 2017, the first gene therapy was approved by FDA [61], raising a great optimism for future genetic disorder treatments.

*When Odysseus reached the island of Lamus near the Telepylus, the city of the Laestrygonians, he was cautious. As one cannot surely know whether he has a genetic predisposition for a disease, so Odysseus could not predict whether some dangerous creatures were living in this unknown land. He decided to keep his ship outside the bay, before exploring the island, while other captains sailed their ships into the harbor and attached them close to one another. But the stay on this island was rather unfortunate. No people were living on it, except for the cannibal giants, who destroyed all the ships but one, hidden outside the bay. Thus, Odysseus lost the big part of his fellow companions, but kept himself and the men on his ship alive, so they continued alone on this long journey, sailing on the open sea*.

## 7. Sixth Stop: Aeaean Island, where Circe lives, and Ethical Issues of Genomic Research

“*Thence we sailed sadly on, glad to have escaped death, though we had lost our*

*comrades, and came to the Aeaean island, where Circe lives—a great and cunning*

*goddess who is sister to the magician Aeetes—for they are both children of the sun*

*by Perse, who is daughter to Oceanus*.” [1]

Along with new, extensive information about individuals, genetics research raised ethical concerns about such experiments. Assuredly, sceptics first considered conspiracies of pharmaceutical and insurance companies, who might use this information for their own benefit. This scepticism is not surprising because genetics may not only predict susceptibility to a certain disease but also may interrogate the history of individual genes and loci under natural selection [62]. Hence, strong ethical and legal foundations were established to protect against gene-based discrimination to minimise the harms and maximise the benefits and confidentiality of genetic studies [63].

The United Nations Educational, Scientific and Cultural Organization (UNESCO) first established a Bioethics Programme in 1993, which was followed by three adaptations: the Universal Declaration on the Human Genome and Human Rights in 1997 [64], the International Declaration on Human Genetic Data in 2003, and the Universal Declaration on Bioethics and Human Rights in 2005 [65].

Protecting genetic information became one of the most important considerations in the development of personalised medicine. Nevertheless, only time will establish public readiness for the day when a genetic test will be as common as a simple blood analysis. Not all individuals will want to follow the new inroads led by medical progress. Indeed, freedom of choice remains one of the basic human rights to be structured into future health systems [29,66]. However, when choosing between life and death, principles are often forgotten in lieu of doing whatever is required to remain healthy and alive.

**Figure 5:**
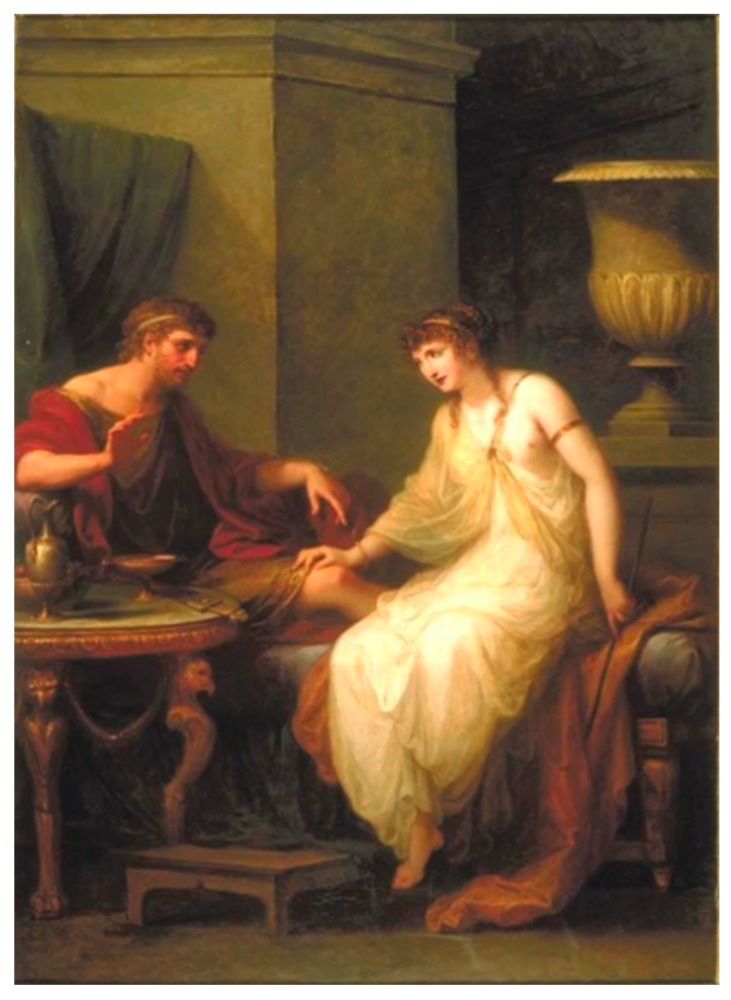
Odysseus and Circe: Angelica Kauffman, 1786. At what point might concerns and angst be disregarded to take advantage of what is offered, notwithstanding fear of the consequences?

*Ethical issues also troubled Odysseus when he met the goddess Circe, a beautiful witch, who was keen on using magic herbs on travellers that passed nearby, transforming them into wild beasts. He was advised by the god Mercury on how to resist her magic potions with a protective talisman and how to provide himself with her loyalty by going to bed with her. Odysseus was deeply in love with his faithful wife Penelope, but did not bother about keeping company with Circe for an entire year (Figure 5). Afterwards, the soldiers became anxious to return and the journey had to continue. Circe kept her promise to help and furthered them towards the House of Hades*.

## 8. Seventh Stop: The Underworld, Personal Genome Project and Cancer Genome Atlas

“*Circe, that great and cunning goddess, sent us a fair wind that blew dead aft*

*and stayed steadily with us, keeping our sails all the time well filled*.”

“*All day long her sails were full as she held her course over the*

*sea, but when the sun went down and darkness was over all the Earth, we got into the*

*deep waters of the river Oceanus, where lie the land and city of the Cimmerians, who*

*live enshrouded in mist and darkness, which the rays of the sun never pierce, neither at*

*his rising nor as he goes down again out of the heavens, but the poor wretches live in*

*one long melancholy night*.” [1]

In 2005, two important projects were launched that impacted personalised medicine. Firstly, the vision of the Personal Genome Project, as a natural successor of the HGP, was to publicly share genome, health and trait data for rapid scientific progress [67]. Initiated by the American geneticist, George McDonald Church, the project was a response to the demand for a highly integrated and comprehensive human genome and phenome datasets to aid research in human functional genomics and systems biology [68]. Currently, it is a network that connects researchers and institutions from the USA, Canada, the UK, Austria and China [67]. The project invites individuals to non-anonymously share their genomic data, traits and cells for free and open research [69,70]. To date, more than 5700 individuals have volunteered (https://my.pgp-hms.org/users) and established the foundation towards the desired goal of 100,000 participants.

Secondly, the Cancer Genome Atlas (TCGA), a collaboration between the National Cancer Institute (NCI) and NHGRI, aimed to catalogue and discover key genomic changes in large cohorts of human tumours [71] and demonstrated the importance of the information acquired through multidimensional genomic analysis for understanding the molecular basis of cancer [72]. The pilot project explored three types of cancers, which were glioblastoma multiforme, and lung and ovarian cancer [73]. Until 2017, it successfully generated comprehensive, multi-dimensional maps for 33 types of cancer and is now concluding [71]. Sharing the value placed on the importance of data sharing with the Personal Genome Project, diverse data from more than 11,000 patients, including clinical information about the patient, metadata about the samples, histopathology images and molecular information, such as gene expression, copy number, SNP genotyping, genome-wide DNA methylation, microRNA profiling and exon sequencing, are publicly available and have thus contributed to more than 1000 studies on cancer [71]. Identifying genes that contribute to functional changes in cells and oncogenic biomarkers allowed for clinical applications and improved the prevention, diagnosis, and treatment of cancer [74]. Finally, it became a model of a successful network and of collaboration for future projects in human health.

Vast innovative collaborations that create networks among researchers around the world are indispensable for generating representative amounts of samples for genomic research progress, which leads to personalised medicine. Integrated knowledge facilitates the path to important discoveries. The aforementioned successful projects provide outstanding examples for the future of research. Ultimately, the goals towards which we are striving are interchangeable worldwide.

**Figure 6:**
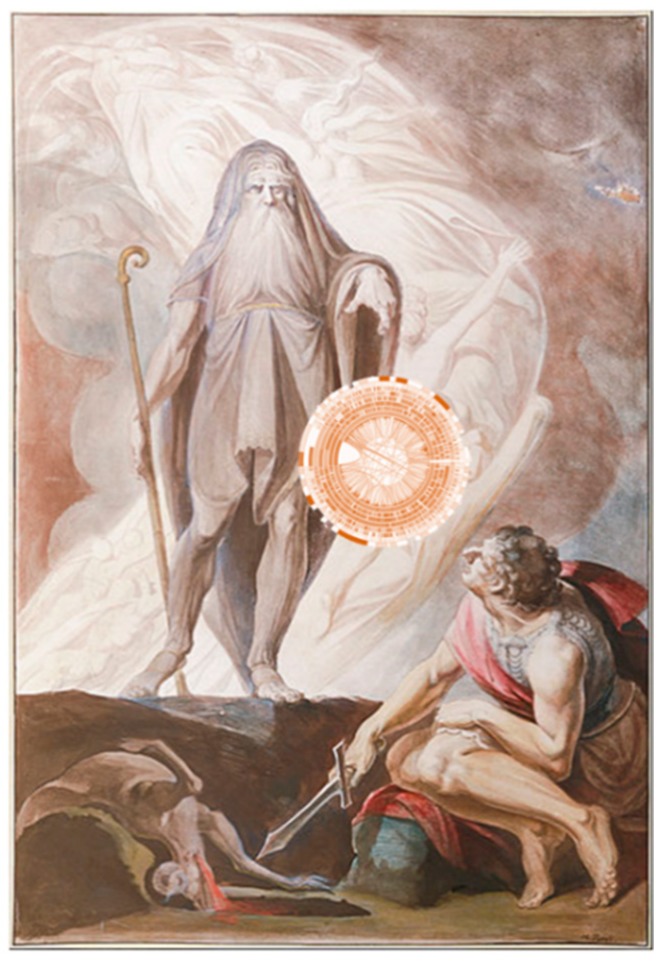
Teiresias and the Cancer Genome Atlas. (Adapted from: https://www.thoughtco.com/).

*In the Underworld, Odysseus was searching for the knowledge that would facilitate his way back home. Thus, he made a sacrifice under Circe’s orders and offered blood to the ghosts with whom he desired to speak. The dead prophet Teiresias (Figure 6) and other ghosts shared important information, which allowed Odysseus to prepare himself for future adventures. He returned to Circe, buried the body of his dead soldier and departed towards the new challenges*.

## 9. Eighth Stop: The Island of the Two Sirens and High-Throughput Technology

“*I had hardly finished telling everything to the men before we reached the island of the*

*two Sirens, for the wind had been very favourable. Then, all of a sudden, it fell*

*dead calm; there was not a breath of wind nor a ripple upon the water, so the men furled*

*the sails and stowed them; then taking to their oars they whitened the water with the*

*foam they raised in rowing*.” [1]

At the beginning of our journey with Odysseus, we discussed the momentous discoveries that led to the rise of the genomics era, which must include one of the most valuable, that of Frederick Sanger. This British biochemist and two-time Nobel prize winner developed a method for DNA sequencing that became the gold standard for the “first generation” of DNA sequencing, and a key technology for the HGP [75].

**Figure 7:**
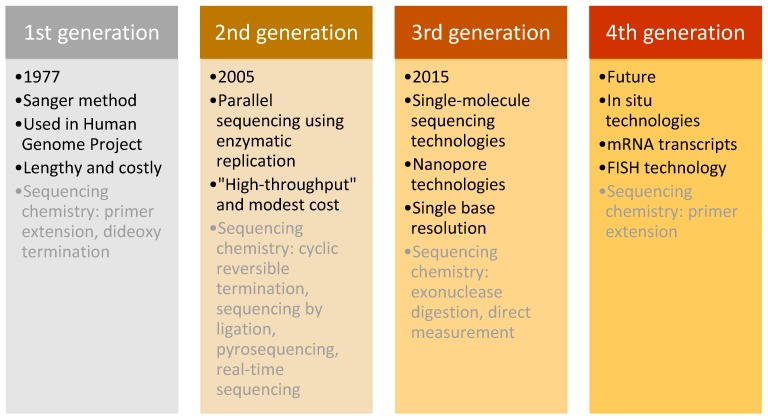
Schematic presentation of the four generations of DNA sequencing.

This technique was used in daily research for more than 30 years, until the arrival of next-generation sequencing (NGS) in 2005 [76]. NGS differed from the Sanger method in its massively parallel analysis, high throughput, and reduced cost [77]. Sequencing methods are classified into three generations plus one future generation, which each have distinguishing characteristics (Figure 7).

Firstly, the “second-generation” sequencing analyses clonally amplified templates that originated from a single DNA molecule, using microfluidic devices and the stepwise addition of four nucleobases. The product of this technology is a high-resolution image that provides accurate sequencing results [78,79]. The three most important technological systems were developed for “second-generation” sequencing, beginning with the 454 Sequencer (currently by Roche), which was the first commercially successful instrument released on the market. It was followed by the Genome Analyzer, developed by Solexa (later purchased by Illumina), which originally could generate 1 G per run. The technology improved and in 2010, Illumina released HiSeq 2000, with an output of 200 G per run. The third fundamental technology that marked the second generation of sequencing was the Sequencing by Oligo Ligation Detection (SOLid) system, made by Agencourt (purchased by Applied Biosystems in 2006) [80]. These technologies progressed over time to provide the most performative, accurate and efficient sequencing possible. They were the first throughput technologies with reduced costs and routine analytic methods for scientific research, which revolutionised genomic research.

Despite the success of second-generation methods, other innovative technologies emerged. The key characteristic of “third-generation” sequencing is the direct reading of a single DNA molecule template without prior processing using enzymatic replication. This feature decreased the bias of previous methods caused by polymerase chain reaction (PCR) amplification. Moreover, these methods increased the read length and decreased the processing time, which provided even higher overall accuracy and enabled rare variant detection at a lower cost [81].

Three categories of “third-generation” sequencing exist: “second-generation” technologies in which single molecules of DNA polymerase are studied, nanopore-sequencing technologies, and the direct imaging of individual DNA molecules using advanced microscopy techniques. The first nanopore DNA sequencer, MinION, commercially available since 2015, was developed by Oxford Nanopore Technologies and provides sequencing for as little as $1000/sample [82].

Finally, the “fourth generation” focuses on examining messenger RNA (mRNA) transcripts using in situ technology (fluorescence in situ hybridisation (FISH)), which allows the detection and genotyping of somatic point mutations with subcellular resolution by directly sequencing nucleic acids in cells and tissue. Furthermore, it employs the conversion of mRNA to localised complementary DNA (cDNA) and thus enables the genotyping of individual transcript molecules. Because expression in single cells may vary substantially from the mean expression detected in a heterogeneous cell population, this method has the potential to revolutionise the field of cancer biology because it enables the mapping of the molecular and cellular tumour environment. Additionally, it is intended to detect defined DNA sequences (such as those that might have undergone a somatic mutation) rather than the complete genotypic sequence from the cell [77,83,84].

Although in situ sequencing has prodigious potential for future research, several technical improvements are needed before it might be broadly applied, specifically sample imaging, the low efficiency of molecular processes, and data handling and interpretation. Should these obstacles be overcome, the method might become a standard for sequencing tissue samples [83].

The “first-generation” sequencing method provided an opportunity for one of the greatest achievements in genomics—the decoding of the human chromosomal genome. This achievement boosted the development of high-throughput technologies that enabled the development of promising tools and led to large-scale studies, which are the engines that further power personalised medicine.

*Passing by the island of Sirens, Odysseus knew that to hear their beautiful singing would lead to inevitable death, as Circe has warned him about the skeletons of unfortunate soldiers, misled by the beauty of the sound, which were lying all around the island, with their flesh still rotting. Thus, he took her advice and plugged the ears of his soldiers but attached himself to the mast, free to hear what no man alive had heard before*.

*With great amount of knowledge, we are free to do things that once seemed impossible. Sequencing that first took more than a decade to be accomplished is now an everyday practice. And Odysseus? His trick, simple and deft, enabled him to bypass the trap and continue on his way unharmed*.

## 10. Ninth Stop: Cliffs of Scylla and Charybdis and Large Gains at a Small Cost

“*Then, we entered the Straits in great fear of mind, for on the one hand was Scylla, and*

*on the other dread Charybdis kept sucking up the salt water. As she vomited it up, it was*

*like the water in a cauldron when it is boiling over upon a great fire, and the spray*

*reached the top of the rocks on either side*.” [1]

No discussion of genetics is complete without considering costs. The final cost of the HGP rose to $2.7 billion for the 13-year endeavour. The WGS of the first “reference” genome included approximately three billion bases, which represented a haploid copy of the human genome, and covered ≈99% of the euchromatic genome, accurate to an error rate of ≈1 event per 100,000 bases [85]. The cost of this project included a wide range of other expenses in addition to sequencing, such as technology development, physical and genetic mapping, model organism genome mapping and sequencing, bioethics research, and programme management. The NHGRI estimated the cost of the potential second “reference” human genome sequencing using the technology available in 2003 as up to $50 million. Though far lower than the initial cost, the use of such methods would still be too costly for routine studies and clinical applications [86]. Based on Sanger’s technique, the diploid genome of the American geneticist Craig Venter was sequenced for approximately $100 million in 2007, and the results were deposited into the public GenBank database [87].

**Figure 8:**
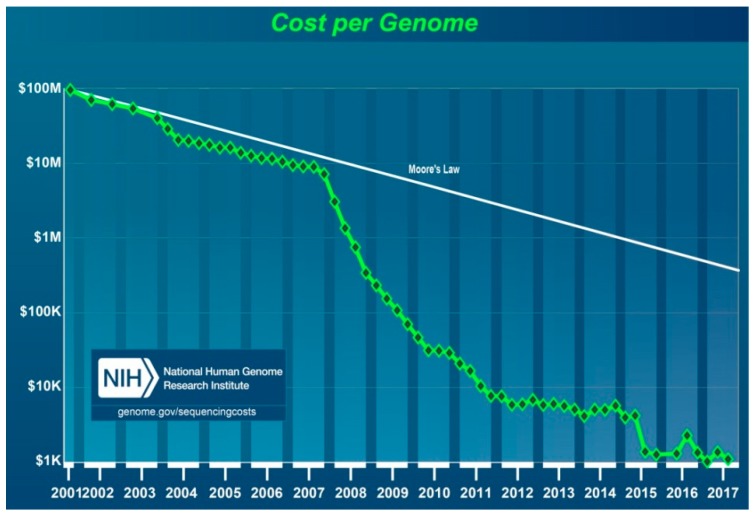
Costs for whole-genome sequencing (WGS). Years 2001–2007 reflect the costs of Sanger sequencing techniques, whilst the data after January 2008 reflect the costs of “second-generation” sequencing. Moore’s Law (in white) is a comparison with the normal long-term trend of progress in the computer hardware industry.

Whilst many ongoing parallel studies contributed to technological progress, the launching of a ground-breaking new project, called the “Advanced Sequencing Technology Awards” or the “$1000 Genome”, set forth a new era in personalised medicine. The NHGRI began this project in 2004 to boost technological progress towards reducing the cost of DNA sequencing for an individual genome to $100,000 in 5 years and $1000 in 10 years. It committed more than $100 million to 50 academic and industrial teams of scientists, including major sequencing companies who, combined, were highly successful. Ultimately, the price of sequencing began to decrease at an accelerated rate after 2007 (Figure 8) [88,89].

Additional incentive was provided by Craig Venter’s foundation, which offered a $500,000 award to stimulate DNA sequencing development with the same final goal of reducing the price of sequencing to $1000 per genome [90]. This award merged with The X Prize Foundation and, in October 2006, formed the Archon Genomics X Prize Foundation, which first announced a $10 million award for technology capable of successfully mapping 100 human genomes in 10 days [91]. The contest began in January 2013, and the prize was intended to be awarded to team(s) able to sequence 100 human genomes with 98% completeness, with an error rate ≈1 event per 1,000,000 bases and a total cost of $1000 per genome, within a time limit extended to 30 days [92]. However, the contest was cancelled in August 2013, due to an unexpectedly high rate of progress in sequencing technologies on the market that required no further encouragement [93].

Indeed, technology progressed much faster than expected. The first NGS of an entire human genome was performed using a Roche 454 Sequencer in 2008. The diploid human genome sequence of James D. Watson was completed in two months using massive parallel sequencing in picolitre-size reaction vessels and was also the “first individual genome to be sequenced for less than $1 million” [94,95]. This advance ushered in a massive reduction in price and the era of next-generation sequencing. Finally, the cost, though still high, was acceptable for routine research and thus permitted the use of a personalised medicine tool necessary for the research and development of new strategies for efficient patient diagnostics and treatment.

**Figure 9:**
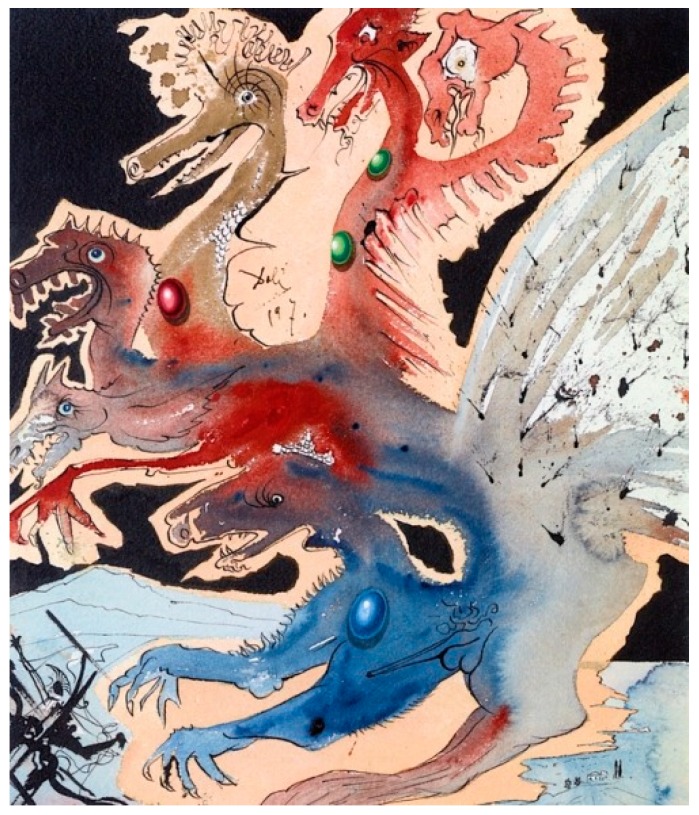
Scylla and Charybdis: Salvador Dali, 1970. There is always a price to pay, but it rests with us to set the limits.

*There is always a price to pay in science, as on the sea. The Sirens were not the only trap at sea that Circe warned Odysseus about. Two monsters, Scylla and Charybdis (Figure 9), were waiting for the sailors and were floating nearby. To avoid Charybdis, a whirlpool that could sink the whole ship, one would get too close to Scylla, a six-headed monster that would eat men alive. They would rather pay the price and continue on their chosen journey than sink after all the effort exerted during their travels*.

## 11. Tenth Stop: The Island of Helios, and the 1000 and 100,000 Genomes Projects

“*When we had passed the rocks, with Scylla and terrible Charybdis, we*

*reached the noble island of the sun-god, where were the goodly cattle and sheep*

*belonging to the sun Hyperion*.” [1]

Many important projects contributed to personalised medicine, several of which were discussed during the recounting of the previous adventures of Odysseus. However, we cannot continue our journey without mentioning the last two, which might share similar titles, but should not be confused with each other.

The 1000 Genomes Project (1KGP) began as a collaboration among research groups from the USA, UK, China and Germany in 2008 and was an extension of the International HapMap Project, which aimed to establish the most extensive catalogue of human genetic variation. Although HapMap was a revolutionary project, it had several limitations, e.g., it only focused on SNPs and no other forms of genetic variations, with an MAF > 0.05. In contrast, the goal of the 1KGP was to establish a resource of at least 95% of the existing variants of human DNA and their haplotype contexts, with frequencies of at least 1% in multiple studied populations. This objective would be fulfilled by sequencing at least 1000 individuals from around the world using next-generation sequencing technologies [96,97,98].

The pilot project was designed to evaluate the strategies of genome-wide sequencing and determine the adequate coverage needed to detect the desired frequencies. It was divided into three steps: (i) WGS of 179 samples with low coverage (2–6×); (ii) deep sequencing of two mother–father–child trios (20–60× coverage); and (iii) exome targeting sequencing of 1000 gene regions in 697 samples (50× coverage). The pilot phase was completed in 2010 and produced robust protocols for whole-genome and targeted sequencing with validated algorithms. Low-coverage sequencing was efficient for detecting and genotyping rare variants and was selected for use in future phases [98,99].

The main project started with a phase two analysis, which generated low-coverage WGS data and exome sequencing for 1092 individuals from 14 populations. It provided a validated haplotype map of 38 million SNPs, 1.4 million insertions/deletions and 14,000 larger deletions, which aided the interpretation of the genetic variants that affect diseases, and provided a publicly available human genome map [100].

The final phase of the 1 KGP was completed in 2015 with the reconstruction of 2504 individual genomes from 26 populations with combinations of low-coverage WGS, deep exome sequencing and dense microarray genotyping [101,102]. Thus, the map included >99% of SNP variants with a frequency of >1% in multiple populations. The analysis of the results demonstrated that most common genetic variants are shared across populations, whilst rare variants are often restricted to closely related populations [103].

The 1KGP was the largest study to sequence the genetic information of such a large number of individuals and its ample variety of genetic information provided the foundation for future disease and population genetic studies. The International Gene Sample Resource is now charged with ensuring future access to and the expansion of the 1 KGP data [98].

In late 2012, the UK Prime Minister launched the 100,000 Genomes Project, which aims to sequence 100,000 whole genomes from approximately 70,000 people recorded in The National Health Service (NHS), England. Led by a public company, Genomics England, it established four main aims: (i) to create an ethical and transparent programme; (ii) to benefit patients and construct a genomic medicine service for the NHS; (iii) to enable new scientific discovery and medical insights; and (iv) to encourage the development of a UK genomics industry [104].

As of early December 2017, more than 41,500 genomes had been sequenced. The project is specifically focusing on rare diseases and cancer because these conditions are strongly linked to genetic mutations. Using longitudinal patient recording, the NHS might correlate a patient’s genome data to phenotypes to thereby aid the understanding of the complex relationship between genes and diseases to not only facilitate the development of a personalised therapy for a patient but also to leave an important legacy for the future [104,105].

Furthermore, since the 1990s, the need for nationwide databases of genetic, clinical and environmental information has been recognised aiming to support the implementation of personalised medicine. The DeCODE project has resulted in the development of a complete database of all the Icelandic population starting at the end of 1990s. More recent efforts include the UK Biobank and the All of Us Research Program in USA.

*When Odysseus arrived on the island with his crew, a huge storm commenced and lasted for a month. Although his crew began to run out of the food, he prohibited them from killing any of cattle grazing nearby because he was warned several times that this would signify the end for all his men. But they did not listen, and killed the biggest animal of the god of the sun, Helios, and all, except Odysseus, feasted. When the storm ended, they continued the journey, but Zeus destroyed their ship and everyone died but Odysseus, who continued floating on the open sea, all alone*.

*Great past and ongoing projects are the proof that in the battle to overcome obstacles in health, scientists, unlike Odysseus, are not alone, but are sailing together with the help of national government towards their Ithaca, better health care for the people*.

## 12. Eleventh Stop: Ogygia, Calypso’s Island, and ‘-omics’ Development

“*Hence, I was carried along for nine days till on the tenth night, the gods stranded me on*

*the Ogygian island, where dwells the great and powerful goddess Calypso. She took me*

*in and was kind to me, but I need say no more about this*.” [1]

We have arrived at the point on our journey at which the foundation laid by all the important projects and discoveries thus far will permit researchers worldwide to perform independent analyses to unravel the complex mechanisms of various diseases. The era of “-omics” studies arose at the beginning of the 21th century on the wings of the technological revolution in rapid throughput technologies.

**Figure 10:**
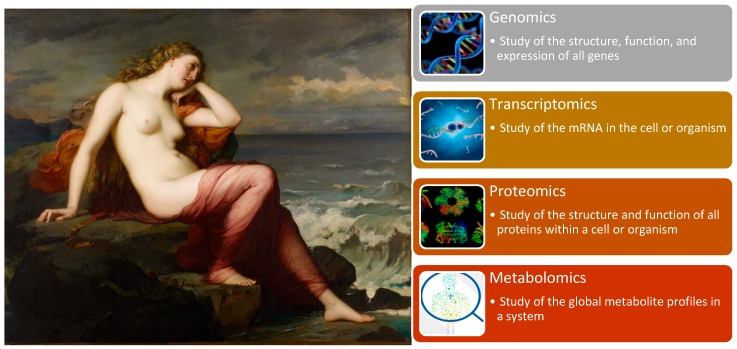
Calypso staring at the four most common “-omics” studies.

“-Omics” technologies are useful tools for detecting genes, mRNA, proteins, metabolites and epigenetic modifications. Genomics, with its well-known GWAS for detecting complex traits and WGS, allows for an understanding of human population and disease genetics, and was previously discussed in the fourth stop of our journey. However, “-omics” technologies provide a holistic view of the molecules that constitute a cell, tissue or organism along with four other branches of this high-dimensional biology: transcriptomics, proteomics, metabolomics (Figure 10) [106].

Transcriptomics emerged with the development of RNA sequencing technologies, which examine an organism’s transcriptome, the sum of all RNA transcripts. Because the RNA profile directly reflects the actual gene activity, it measures the expression of an organism’s genes and provides information regarding how genes are regulated [107]. In combination with genomics, RNA sequencing data provide a unique perspective on genetic functional variants and a deeper understanding of the cellular mechanisms of transcriptome variation [108].

Proteomics is the large-scale study of proteomes, which encompasses the aggregate of all expressed proteins within a cell, system or organism, and their structure and function. This field represents the next step after genomics and transcriptomics research for examining biological systems. For a long time, protein research was restricted due to technological limitations, which were overcome by the arrival of new mass spectrometry technologies. Proteomics aims to elucidate the functional role of proteins through their pathways and networks. It reflects gene and environmental dynamics and thus is closely related to functional genomics [107,109]. Proteomics remains a complex field because of the vast dynamics of proteins; however, it is a promising field for the discovery of new biomarkers because most commonly it is proteins that are affected during disease or a disease response. With future progress in technology, we will be able to achieve greater advances in the field and improve research on new therapeutic targets [110].

Finally, metabolomics is the last of the basic “-omics” technologies. It is the study of the chemical processes concerning metabolites, which thus reveals the energy status and metabolism of an organism. It reflects the changes in the transcriptome and proteome. Though it is the smallest of the four main “-omics” branches, it is the most physically and chemically complex [106,107].

Another emerging field is epigenetics modifications, which include heritable modifications of the gene expression regulation that are not due to nucleotide changes of DNA sequence. These modifications include the DNA methylation, histone modification and non-coding RNA (ncRNA)-associated gene silencing [111]. High throughput screening assays are available today for the study of epigenome, while large-scale projects aiming to the development of public datasets of epigenetic modifications exist. The Encyclopedia of DNA Elements (ENCODE) aims to identify all regions of transcription, transcription factor association, chromatin structure and histone modification in the human genome sequence [112]. The Natioanal Institutes of Health (NIH) Roadmap Epigenomics Mapping Consortium is developing a public resource of human epigenomic data aiming to support basic and medical research related to the epigenetics field [113].

Systems biology is using “-omics” studies for detailed examinations of organisms and their molecular phenotypes. It facilitates the generation of complex molecular pathways that might only be revealed through a comprehensive approach. The accumulating knowledge from “-omics” research provides vast possibilities for the targeted treatment of diverse medical conditions, the discovery of novel possible biomarkers for diseases diagnostics and prognostication, and the transformation of conventional symptom-orientated medicine to preventive and personalised health care.

Such studies cannot bring immediate change but rather require years to transform discoveries into practical applications. Thus, the “-omics” era is passing through a long, calm period with much progress but no substantial perturbations thus far.

*The calm period was there also for Odysseus, who settled in Ogygia, on the island of a nymph called Calypso. She fell in love with the hero and wanted to have him as her husband. For seven years, they lived together on the island, until the goddess Athena interrupts the story. Finally, Calypso had to let her lover go, and helped build a raft that would take him towards the final stop of his long journey, before reaching home*.

## 13. Twelfth Stop: Scheria, the Island of the Phaeacians and Alliances of Personalised Medicine

“*Thereon he floated about for two nights and two days in the water, with a heavy swell*

*on the sea and death staring him in the face; but when the third day broke, the wind fell*

*and there was a dead calm without so much as a breath of air stirring. As he rose on the*

*swell he looked eagerly ahead, and could see land quite near. Then, as children rejoice*

*when their dear father begins to get better after having for a long time borne sore*

*affliction sent him by some angry spirit, but the gods deliver him from evil, so was*

*Odysseus thankful when he again saw land and trees, and swam on with all his strength*

*that he might once more set foot upon dry ground*.” [1]

This account has consistently shown that only with mutual assistance, collaboration and scientific exchange can science achieve its goals of improving health systems through novel strategies of P4 medicine—predictive, preventive, personalised, and participatory. Currently, we are apparently wedged in a transitional period of personalised medicine, a time during which everyone is discussing it and researching its applications, but without a sense of substantial change during a simple visit to the doctor. This dissociation is why collaborations between physicians, public and private researchers and politicians are critical to allow the circulation of knowledge from research institutes supported by industry to hospitals and governments. This sequence will lead to more rapid advances and progressive changes for current health systems that will directly benefit patients.

Healthcare experts from across Europe are gathering at diverse international conferences, consortia and societies to accelerate the development and delivery of personalised medicine and diagnostics. Of these, firstly, the Santorini conference series, Systems Medicine and Personalised Health and Therapy (http://santoriniconference.org/), which was initiated in 2002 and aimed to assemble scientists from genetic and biochemical backgrounds, was dedicated to personalised medicine [114]. It is the oldest international conference in the field of pharmacogenomics and personalised medicine, and is held every two years [114,115,116,117,118,119,120,121]. One of the important outgrowths from the Santorini conference series was the formation of the European Society of Pharmacogenomics and Personalised Therapy (ESPT), founded in 2011 (https://esptnet.eu/). It soon became one of the leading organisations in the field of pharmacogenomics and personalised medicine, and aims to integrate multidisciplinary approaches with research and transform them into clinical benefits for professional training and education of the general public in all areas of human pharmacogenomics, clinical pharmacology, laboratory medicine and personalised medicine [122], extending the actions of the Santorini conference series. The organisation connects research groups with corporate members and national societies to enhance the scientific basis for the quality of patient diagnosis and therapy through efficient communication with ESPT members, other healthcare providers, regulators and the public to disseminate information and educate them regarding outstanding scientific and educational achievements [122].

Secondly, the European Alliance for Personalised Medicine was launched in 2012 to change healthcare using personalised approaches towards each patient (https://www.euapm.eu/). By developing case studies, organising workshops, education, training and communication, they attempt to increase awareness and an understanding of personalised medicine and have an important impact on the progress of this field, through increased research and development [123].

**Figure 11:**
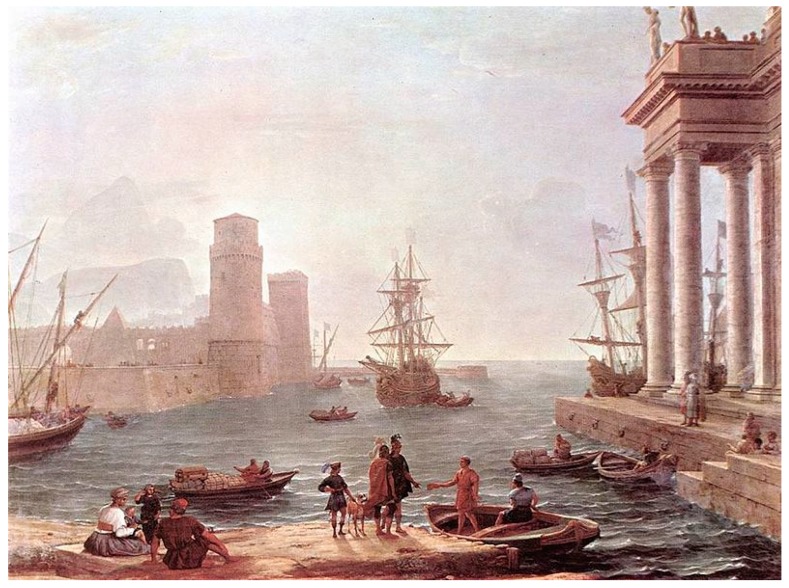
Departure of Ulysses from the Land of the Phaeacians: Claude Lorrain, 1646.

*Even this time, the journey did not go well, for the god, Poseidon, was still angry with Odysseus for blinding his son, so he sent the winds and storm over the sea. But Odysseus always had allies on his side. This time, the goddess Ino gave him her magic veil to help him reach Scheria, where Nausica, daughter of the King Alcinous, found him. Odysseus was invited to dinner, where he told his long story about the journey from Troy towards home. The Phaeacians, moved by his tale, offered him treasures and provided him safe passage back to Ithaca (Figure 11)*.

## 14. Thirteenth Stop: Ithaca and the Future of Personalised Medicine

“*The ship bounded forward on her way as a four in hand chariot flies over the course*

*when the horses feel the whip. Her prow curvetted as it were the neck of a stallion, and*

*a great wave of dark blue water seethed in her wake. She held steadily on her course,*

*and even a falcon, swiftest of all birds, could not have kept pace with her. Thus, then,*

*she cut her way through the water, carrying one who was as cunning as the gods, but*

*who was now sleeping peacefully, forgetful of all that he had suffered both on the field*

*of battle and by the waves of the weary sea*.” [1]

**Figure 12:**
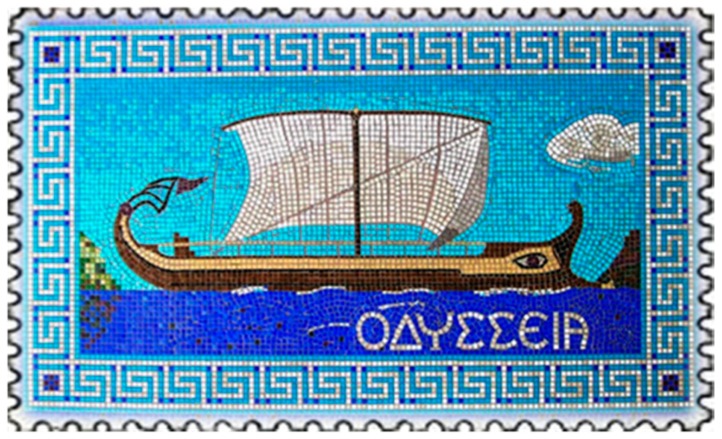
The journey to Ithaca. (Adapted from: http://santoriniconference.org/). Ithaca–Konstantinos Kavafis (1863–1933).

*Odysseus managed to overcome all the obstacles to reach the Ithaca (Figure 12) he was longing for, yet the story did not conclude. Angry suitors were waiting, for faithful queen Penelope must finally choose to marry one of them. And Odysseus must fight them, to save his family, to make peace and order on his island again. He has not tired of the journey however, because he knows that a new, brighter future awaits*.

A new, brighter future also awaits personalised medicine.

New extraordinary findings are reminding us every day that we are on the right path. With “-omics” information, we are illuminating new hypothesis on molecular mechanisms of disease, and translating them into advice for disease prevention and improved diagnostics and therapeutics. Moreover, researchers are now realising that genotyping information could give us many new answers on disease mechanisms, just by using the causal connections between identified variants and disease pathways [124]. With holistic “-omics” perspective, looking at genes, proteins, gut microbes and metabolic markers, practitioners will be able to see how these elements of health change as people progress through the course of their lives.

Pharmacogenomics studies using GWAS have discovered numerous rare and common variants in different populations that control individual drug responses. This led also to more precision trials, because drug candidates can be tested in more targeted subpopulations [125].

Finally, the new era in medicine started in 2017 with the first FDA-approved gene therapies for treating acute lymphoblastic leukemia (Kymriah), large B-cell lymphoma (Yescarta) and an inherited form of childhood blindness (Luxturna) [126]. Moreover, for the first time, scientists demonstrated the possibility of treating Duchenne muscular dystrophy in large animals, by delivering gene editing components that can restore affected protein [127]. All these new therapies are giving hope and posing questions. Will there be a day when we will no longer treat a disease, but our genome?

Until then, we must continue to confront the challenges posed by genomics; moreover, we must continue to compile genomic variants, each of which accounts for a small percentage of disease susceptibility. Each small discovery represents a step closer to a day when all the efforts of research and industry will be combined into diagnostic and therapeutic tools that will revolutionise medicine in favour of patients and healthcare providers and regulators.

*When you set out on your journey to Ithaca,*

*pray that the road is…*

*full of adventure, full of knowledge…*

*That the summer mornings are many, when,*

*with such pleasure, with such joy you will enter ports seen for the first time…*

*Always keep Ithaca in your mind*.

*To arrive there is your ultimate goal…*
